# Laryngeal edema following remimazolam-induced anaphylaxis: a rare clinical manifestation

**DOI:** 10.1186/s12871-023-02052-w

**Published:** 2023-03-29

**Authors:** Xiawei Hu, Yaning Tang, Xiangming Fang

**Affiliations:** Department of Anesthesiology, The First Affiliated Hospital, Zhejiang University School of Medicine, Hangzhou, Zhejiang China

**Keywords:** Remimazolam, Anaphylaxis, Sedation

## Abstract

**Background:**

Remimazolam is an ultra-short-acting intravenous benzodiazepine, which has been used as sedative/anesthetic in procedural sedation and anesthesia. Although peri-operative anaphylaxis due to remimazolam has been reported recently, the spectrum of the allergic reactions is still not fully known.

**Case presentation:**

We describe a case of anaphylaxis following remimazolam administration in a male patient undergoing colonoscopy under procedural sedation. The patient presented complex clinical signs including airway changes, skin symptoms, gastrointestinal manifestations and hemodynamic fluctuations. Different from other reported cases, laryngeal edema was the initial and main clinical feature of remimiazolam-induced anaphylaxis.

**Conclusions:**

Remimazolam-induced anaphylaxis has a rapid onset and complex clinical features. This case reminds anesthesiologists should be particularly alert to the unknown adverse reactions of new anesthetics.

**Supplementary Information:**

The online version contains supplementary material available at 10.1186/s12871-023-02052-w.

## Background

Of all wide spectrum of adverse drug reactions, peri-operative anaphylaxis is undoubtedly the most disconcerting event to anesthesiologists. It is a life-threatening reaction characterized by acute onset of symptoms involving different organ systems and requiring immediate medical intervention [1]. Remimazolam besylate, a novel ultrashort-acting benzodiazepine, has recently been approved for clinical use as a general anesthetic. Although peri-operative anaphylaxis due to remimazolam has been gradually ascertained and reported [2–5], the spectrum of its allergic reactions is still not well known. Here, we describe a case of anaphylaxis caused by remimazolam, which presented with complex clinical signs, including airway changes, skin symptoms, gastrointestinal manifestations and hemodynamic fluctuations. Written informed consent was obtained from the patient for the publication of this case report.

## Case presentation

A 41-year-old male (height, 165 cm; body weight, 63 kg) was scheduled for colonoscopy review. He had no history of any remarkable disease, symptom, or drug allergy. He had undergone gastroscopy and colonoscopy with unknown sedative drugs in the physical examination institution two years ago, and colorectal polypectomy by endoscopic mucosal resection by sedation with propofol half a year ago in our hospital. Deep sedation with monitored anesthesia care was induced and maintained with the administration of propofol and alfentanil without any problems.

**Fig.1:**
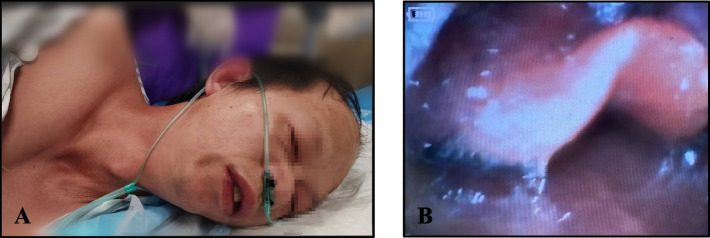
Clinical presentation when peri-operative anaphylaxis occurred showing significant flushing on face, neck and chest, a depression in the suprasternal, periorbital edema and lip swelling **A**, and epiglottic edema under visual laryngoscope **B**

Before anesthetics administration, the standard vital signs were monitored and were as follows: non-invasive blood pressure (NIBP), 116/69 mmHg; heart rate, 85 bpm; and SpO_2_, 100%. After starting oxygenation at 2 L/min by nasal oxygen cannula, we administered 10 mg of remimazolam (0.15–0.2 mg/kg) (Yichang Humanwell Pharmaceutical Co., Ltd, China) for introduction. Within 1 min, the patient presented audible laryngeal stridor with marked depression of suprastemal fossa. Immediately, we observed a large area of erythema on his face, neck and chest. We further observed periorbital edema and lip swelling (Fig. 1A, video in Appendices). With a stethoscope, we listened to the breath sounds of both lungs, but these were too light at that moment. Following SpO_2_ drop to 91%, we performed a jaw thrust maneuver, manual ventilation and 100% oxygen. Spontaneous breathing continued, but the SpO_2_ level fluctuated between 85–95%. Considering the skin features, we speculated the presence of laryngeal edema, causing severe inspiratory dyspnea. Epiglottic edema was observed under visual laryngoscope, which confirmed our hypothesis (Fig. 1B). Copious oral secretions were noted, requiring aggressive suctioning. In parallel with respiratory compromise, 1 min after introduction, NIBP was stable at 100/64 mmHg, but after approximately 3 min, NIBP dropped to 77/47 mmHg, and heart rate increased to 95 beats/min. As anaphylaxis was strongly suspected based on the clinical presentations, we intravenously administered 25 μg of adrenaline, but hemodynamics did not change drastically. We then administered 25 μg of intravenous adrenaline repeatedly (total 50 μg) and 2000 mL of crystalloid, which improved NIBP to 116/79 mmHg. Despite improvement in the patient’s blood pressure, SpO_2_ still fluctuated between 81–87%. We initiated arterial blood pressure monitoring with cannulation of the left radial artery. Blood gas test results showed a significantly elevated PCO_2_ of 104 mmHg. Considering the patient had previously used propofol without adverse reactions, we intravenously administered 12 mg propofol, combined with 4 mg vecuronium, and inserted a laryngeal mask airway to facilitate mechanical ventilation. Consequently, SpO_2_ rose gradually to 100% and the patient’s vital signs were stable. After discussion, the physician was allowed to proceed with colonoscopy examination, which revealed a large amount of yellow rectal discharge, and this was consistent with the diarrhea caused by allergy. Furthermore, for a definitive diagnosis, the patient’s intestinal mucosa was biopsied and sent for histopathologic examination.

**Fig. 2:**
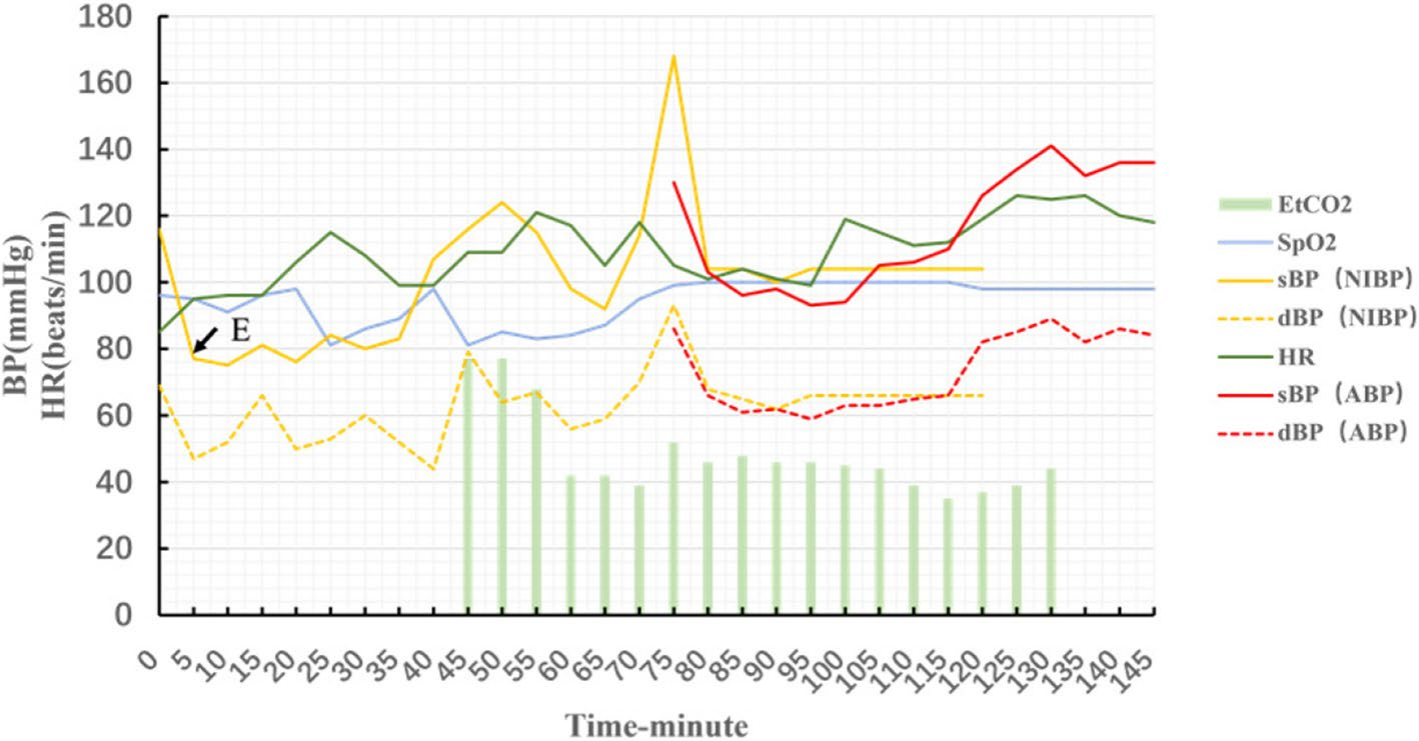
The patient’s vital signs during the anaphylactic episode. BP: blood pressure; NIBP: non-invasive blood pressure; ABP: intra-arterial blood pressure; HR: heart rates; sBP: systolic blood pressure; dBP: diastolic blood pressure; EtCO2: end-tidal carbon dioxide; E, adrenaline. The arrow indicates the time of initial adrenaline administration

The whole process lasted 2 and a half hours. The patient naturally awoke and was hemodynamically stable, but complained of eye photophobia and tearing. After 30 min of observation, the patient was returned to the general ward. The change in the patient’s vital signs during the episode is shown in Fig. 2 and the results of blood gas test are shown in Table 1.

**Table 1:** Arterial blood gas analysis results performing before mechanical ventilation, after mechanical ventilation and in PACU

	**Before MV**	**After MV**	**In PACU**
**pH**	7.054	7.218	7.331
**pCO2(mmHg)**	104	63.9	46.9
**pO**_**2**_** (mmHg)**	75.7	274	154
**sO2 (%)**	86.1	100.2	99.8
**cNa + (mmol/L)**	144	143	142
**cK + (mmol/L)**	2.9	3.5	3.6
**cCa2 + (mmol/L)**	1.01	1.1	0.90
**cGlu (mmol/L)**	8.7	7.6	4.7
**cLac (mmol/L)**	1.2	0.6	0.7
**Hct, c (%)**	48.6	47.9	45.2
**HCO3- (mmol/L)**	29.0	26.0	24.7
**BE(B)**	-1.4	-1.7	-1.2
**THbc (g/L)**	150	156	147

*MV* Mechanical ventilation, *PACU* Postanesthesia care unit

**Fig. 3:**
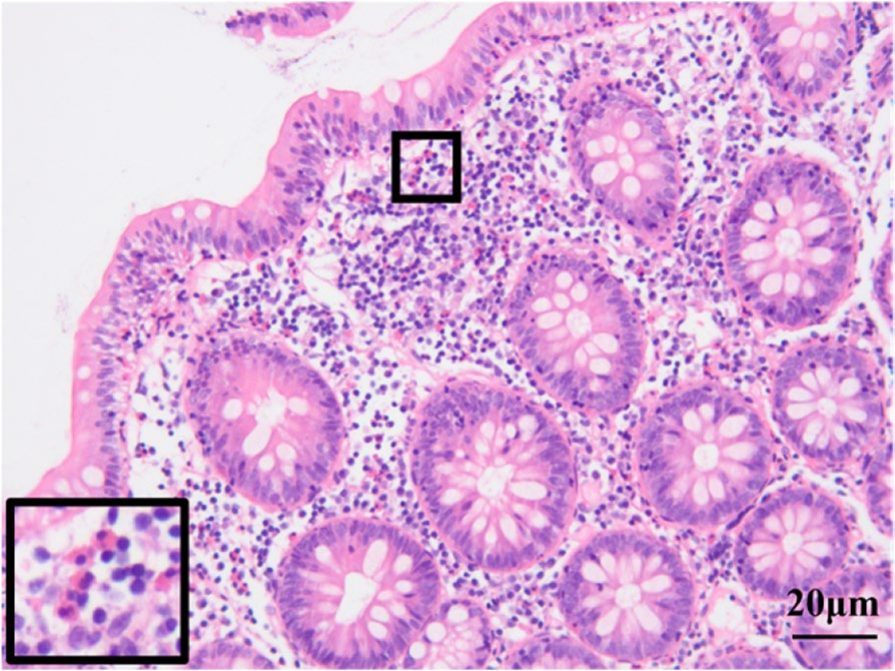
Representative image of ascending colon mucosa by H&E staining (magnification: 40X), showing eosinophils infiltration, achieve to 70/HPF in the area with high cell density. Inset box shows the higher magnifications of eosinophils infiltration. HPF: high power field

**Fig. 4:**
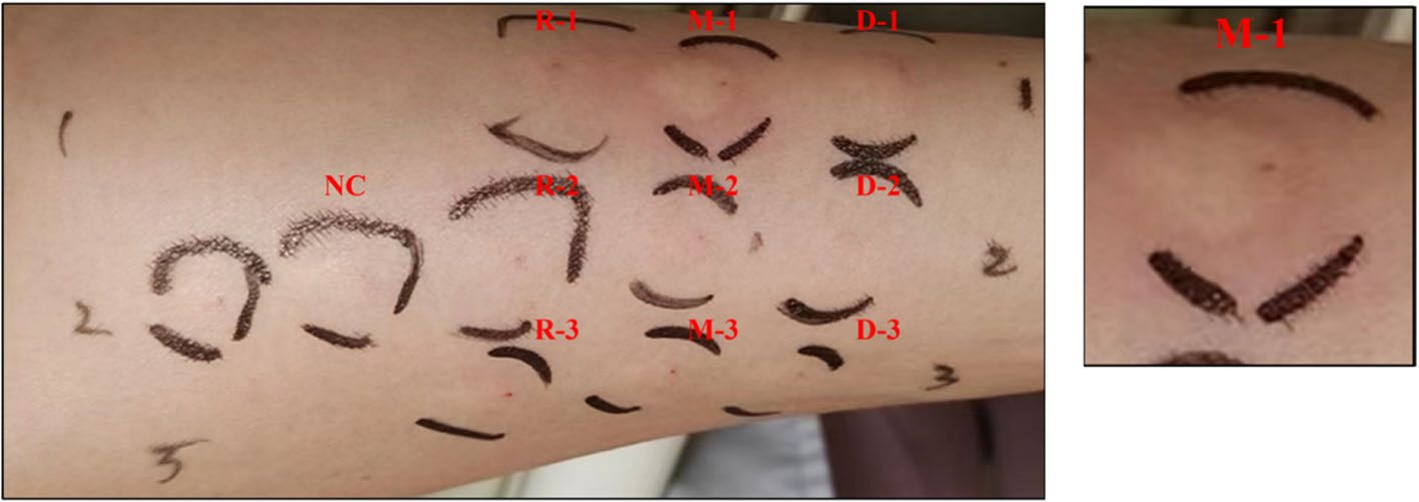
The intradermal test shows respective reactions of remimazolam, midazolam, dextran 40 (left) and a positive reaction to midazolam (right). R-1, 2, 3 means remimazolam solution (1 mg/ml), diluted 1:10 (0.1 mg/ml) and 1:100 (0.1 mg/ml) in saline respectively; M-1, 2, 3 means midazolam solution (1 mg/ml), diluted 1:10 (0.1 mg/ml) and 1:100 (0.1 mg/ml) in saline respectively; D-1, 2, 3 means dextran 40 solution (0.6 g/ml), 1:10 (0.06 g/ml) and 1:100 (0.006 g/ml) in saline respectively

Histopathologic examination showed eosinophil infiltration in the mucosa stroma of the ascending colon, with about 70/HPF in the dense area (Fig. 3). Four weeks after the event, the patient underwent skin tests to confirm the causative allergic agent. The intradermal tests for remimazolam, midazolam, dextran 40 were performed with 0.1 ml of each sample. Remimazolam and midazolam were first diluted with saline to 1 mg/ml and further again with saline to a ratio of 1:10 and 1:100. Interestingly, a markedly positive reaction was recorded at the test site with midazolam (erythema of 16 × 11 mm and swelling of 10 × 8 mm), but not with remimazolam and dextran 40 (Fig. 4).

## Discussion and conclusions

Remimazolam besylate, a new ultrashort-acting GABA_A_ receptor agonist, was approved in 2020 for general anesthesia and/or procedural sedation worldwide [6]. Owing to its advantageous characteristics of fast onset of action and rapid recovery without significant cardiovascular or respiratory depression, remimazolam has been widely used for pre-procedural sedation for endoscopy outside the operating room [7]. Herein, we demonstrated a case of anaphylaxis caused by remimazolam in ambulatory endoscopy center.

Diagnosis of anaphylaxis is mainly based on history and clinical criteria for organ system involvement [8]. There is various clinical evidence supporting anaphylaxis caused by remimazolam. First, typical clinical manifestations were recorded following remimazolam administration. Airway changes were the main and first symptoms observed, including dyspnea with laryngeal stridor and subsequent hypoxia. In addition, skin changes presented with a sudden flush on patient’s face and chest, edema around the orbit and lips, photophobia and tears. The patient also presented with diarrhea, suggesting gastrointestinal involvement. Second, NIBP showed no change when dyspnea first happened. Hypotension occurred approximately 3 min after administration of remimazolam, indicating that respiratory compromise preceded hemodynamic fluctuations. Last but most importantly, a pathological examination of the mucosa stroma in the colon showed extensive infiltration of eosinophils, which strongly indicated anaphylaxis.

Following the clear diagnosis of anaphylaxis, identification of the causative agent is critical for the patient. According to the pharmaceutical label indication, the remimazolam solution contains lactose monohydrate, hydrochloric acid, sodium hydroxide, remimazolam, and dextran 40. Among them, remimazolam itself and dextran 40 are the most likely to be the causative allergens. Remimazolam was a new agent but it has a similar chemical structure to midazolam. Thus, we performed skin testing with midazolam, remimazolam and dextran 40. Interestingly, in our case, the intradermal tests only presented positive reaction to midazolam, but not remimazolam nor dextran 40. Nevertheless, the methodology for remimazolam skin tests has not been standardized. We used 1:10 and 1:100 remimazolam dilutions with saline. To maintain consistency with the diluted concentration of remimazolam, we also used 1:10 and 1:100 midazolam dilutions. It is still questionable whether the concentration of these two drugs diluted in equal proportion is comparable. Therefore, there was a possibility of false-negative result of remimazolam. Although this patient was unable to provide the record of sedative agents when he underwent gastroscopy and colonoscopy two years ago, we firstly presume that remimazolam itself as the likely allergen in this case. This presumption is also declared in other case reports, for example, the skin prick test result of Tsurumi et al [2]. indicated that both remimazolam and midazolam showed positive reactions. Intradermal tests by Hasushita et al [5]. yielded positive reaction to remimazolam but not midazolam. On the other side, the diluent of dextran 40 was from.

“Dextran 40 and Glucose Injection” 500 ml (Kelun Pharmaceutical Co., Ltd, China), containing 30 g dextran 40 and 25 g glucose. Dextran 40 is known to cause anaphylaxis and the anaphylactoid symptoms is non-IgE-mediated [9]. As expected, the skin test result of dextran 40 showed negative. In addition, five case series reported by Kim et al [4]. maintained dextran 40 as the cause of anaphylaxis rather than remimazolam. Thus, we can not absolutely rule out the possibility of dextran 40 causing anaphylactoid symptoms.

There have been several case reports of anaphylaxis caused by remimazolam recently. Almost all cases occurred in the induction period of general anesthesia with endotracheal intubation. Severe hypotension was the initial and main evidence for anaphylactic shock. For example, one case report described hemodynamic collapse within 2–3 min after tracheal intubation in 3 cases [4]. Hasushita et al [5]. found that blood pressure dropped sharply and skin erythema occurred 6 min after tracheal intubation. Subsequently, this patient developed cardiac arrest. Fortunately, our case presented with anaphylaxis during the induction episode of procedural sedation. Airway compromise and skin signs could be found initially, which led to our suspicion of peri-operative anaphylaxis. Therefore, when hypotension and tachycardia presented, we administered an intravenous bolus of adrenalin for a prompt response. As expected, early use of adrenaline combined with the muscle relaxant vecuronium quickly stabilized the patient’s hemodynamics and relieved airway obstruction. In comparison with our case, anaphylaxis in other patients was discovered rather late – more than 3 min or even 6 min, and hypotension and tachycardia were the prominent presenting features. It was speculated that the anaphylaxis induced by remimazolam would present with isolated cardiovascular features, since raised airway pressure or oxygen desaturation was not reported. We also considered the possibility that mechanical ventilation following neuromuscular blocking agents partly masked the airway problem. Altogether, comprehensively understanding the variations in presenting clinical signs of remimazolam-induced anaphylaxis between different patients can help anesthesiologists diagnose and deal with anaphylactic situations as early as possible in their practice.

Importantly, we must underline that the laryngeal edema was the predominant clinical sign in this event. Visual laryngoscopy revealed epiglottic edema, suggesting that laryngeal edema contributed to the upper the upper airway collapse. Although auscultation was unclear – such that no abnormal breathing sound could be collected, we cannot rule out bronchospasm. After all, resuscitation with manual ventilation and oxygen was unsuccessful, and led to carbon dioxide retention. Arguably, we should have performed invasive arterial monitoring and blood gas analysis earlier to guide us to provide timely further treatment, such as the tracheal intubation to facilitate mechanical ventilation. Thankfully, prolonged hypoxemia was corrected by laryngeal mask intubation followed by the administration of muscle relaxant.

The limitation of our study is that because of lack of reagents, we did not conduct the serum tryptase assay, which is helpful for diagnosis of anaphylaxis. Despite this shortcoming, pathological evidence showed eosinophil infiltration in the intestinal mucosa, supporting the diagnosis. Eosinophiles have been associated with allergic disease pathogenesis for over 100 years via release of several cytokines and other cells involved in inflammation, amplification and regulation of localized immune responses [10].

Our experience based on this case is that remimazolam-induced anaphylaxis has a rapid onset, accompanied by airway, skin, gastrointestinal and hemodynamic changes. Thus, anesthesiologists should be particularly alert to the unknown adverse reactions of new anesthetics.

## Supplementary Information

**Additional file 1.** Supplementary: the video for the patient’s airway manifestations following remimazolamadministration under procedural sedation. 

## Data Availability

All data related to this case report are contained within the manuscript.
