# Supplementary material for: Laryngeal edema following remimazolam-induced anaphylaxis: a rare clinical manifestation
Source: BMC Anesthesiol. 2023 Mar 29;23:99. doi: 10.1186/s12871-023-02052-w (PMC10053874; doi:10.1186/s12871-023-02052-w)
Supplement: Supplementary file 1 — Additional file 1. Supplementary: the video for the patient’s airway manifestations following remimazolamadministration under procedural sedation. [file 12871_2023_2052_MOESM1_ESM.pptx]

## Slide 1
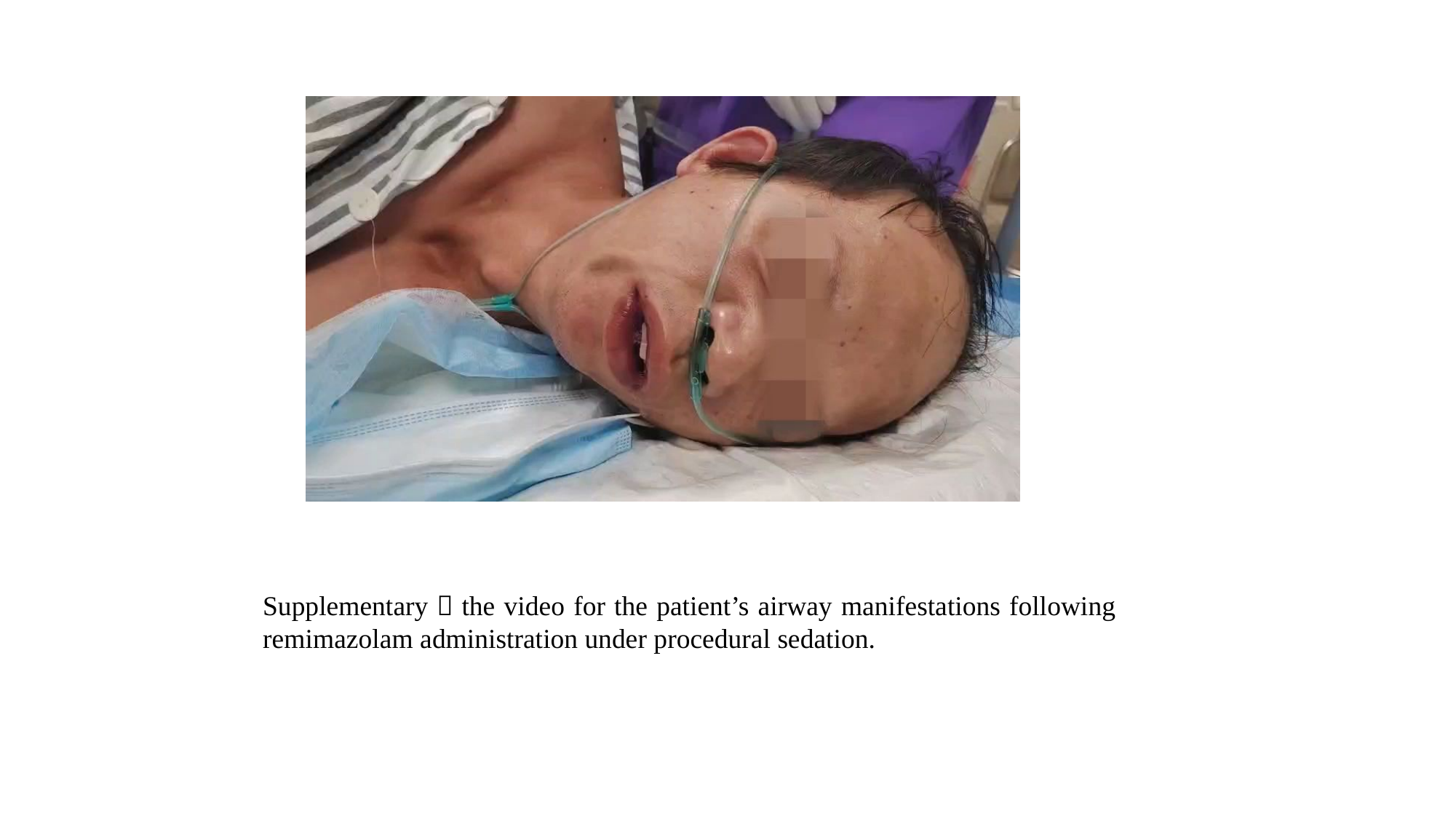

Supplementary：the video for the patient’s airway manifestations following remimazolam administration under procedural sedation.
